# Reply to “Limitations of the Digestible Indispensable Amino Acid Score (DIAAS) and Choice of Statistical Reporting. Comment on “A Comparison of Dietary Protein Digestibility, Based on DIAAS Scoring, in Vegetarian and Non-Vegetarian Athletes.” *Nutrients* 2019, *11*, 3106”

**DOI:** 10.3390/nu12041184

**Published:** 2020-04-23

**Authors:** Carol S. Johnston, Heidi Lynch, Christopher Wharton

**Affiliations:** 1 College of Health Solutions, Nutrition Program, Arizona State University, 550 N. 3rd St., Phoenix, AZ 85004, USA; 2 Kinesiology Department, 3900 Lomaland Dr., Point Loma Nazarene University, San Diego, CA 92106, USA; hlynch@pointloma.edu; 3 College of Health Solutions, Voluntary Radical Simplicity lab, Arizona State University, 550 N. 3rd St., Phoenix, AZ 85004, USA; christopher.wharton@asu.edu

We appreciate the critique of our paper (Ciuris et al. [1]) by Genoni et al. [2]. Our research group has a track record of investigating the benefits of vegetarian nutrition, such as demonstrating that vegetarian diets promote healthful mood states [3,4] and improve acid-base balance [5], along with, developing an evidence-based vegetarian food guide [6]. However, our research also suggests that lower protein intakes among vegetarians may pose challenges for some health outcomes; specifically we have linked lower protein intakes in vegetarians to lower bone mineral density [7] and strength [8]. To follow on the latter finding, we recently conducted a randomized trial to examine the impact of supplemental plant protein on strength in sedentary vegetarians [9]. Participants were randomized to a mung bean food supplement (18 g protein/d) or control bar (4 g protein/d). (Note that both supplements were vegan friendly.) After 8 weeks, the average percent change for grip, flexor, and extensor strength differed between groups (+2.9 ± 7.2% and −2.6 ± 7.3%, respectively for the mung bean group versus the control group; *p* = 0.05). These results suggest that supplementary protein (plant-based) may improve strength in the absence of exercise, a finding that was independent of lean mass [9]. In this trial, we also reported that the mean grip strength of participants at baseline (25.9 ± 7.3 kg) was significantly below the reference value for North American females (31 kg; *p* < 0.001), which may further indicate protein inadequacy in this population. 

Genoni et al. [2] stated three concerns with our paper. First, they suggested that the absence of fruit and vegetable foods in our Digestible Indispensable Amino Acid Score (DIAAS) analysis skewed the results since these foods contribute substantially to dietary protein. For our analyses, we utilized the food categories presented in the 2011 report of the Food and Agriculture Organization Expert subcommittee [10]. To our knowledge, the true ileal amino acid digestibility is not known for fruits and vegetables aside from legumes, which were utilized in our analyses (see Table A2, [1]). We acknowledged in our paper “the DIAAS spreadsheet utilized in this research was not extensive, as it was limited by the availability of true ileal digestibility values that have been derived for foods.” Note that the Dietary Reference Intake for protein, released in 2005 by the Food and Nutrition Board of the Institute of Medicine [11], refers only to grain (wheat) and legumes (chickpea) when discussing non-animal dietary protein quality. Hence, to date, data are not available to assess the usability of plant proteins aside from the grain and legume categories. Although Genoni et al. cite the Papier et al. paper [12] in support of their argument, these authors list legumes, pulses, protein alternatives (soya, tofu), nuts, cheese, yogurt, milk, plant milk, and eggs—but not fruits and vegetables—as the major protein foods for vegetarians. 

Secondly, Genoni et al. are critical of our use of the DIAAS in athletes stating that DIAAS analyses do not consider the metabolic demand for protein in the context of exercise. Citing Burd et al. [13], Genoni et al. argue that, in comparison to a sedentary state, physical activity (specifically, strength training) ‘lowers’ the amount of dietary protein required to support muscle synthesis since amino acid sensitivity of muscles is enhanced. Yet, Burd et al. clearly acknowledge that endurance exercise appears to have the opposite effect on the total daily dietary protein requirement for athletes compared to sedentary people, with regular endurance exercise training placing “more demand on dietary protein,” a phenomenon that is accentuated by intensity and exercise duration [13]. Our athletes were competitive endurance athletes (triathletes, runners, or cyclists); hence, if the assertions of Burd et al. are indeed true, our DIAAS analyses may have underestimated the protein needs of these athletes. 

Finally, Genoni et al. suggest that our statistical analyses were “flawed” for comparing strength differences between diet groups since we did not control for age, gender, energy intake, and lean mass. The stated purpose of our study was to “analyze dietary protein availability using the DIAAS method and to relate available protein to muscle mass and strength.” We demonstrated significant relationships between available protein and muscle mass (r = 0.541) and available protein and peak torque (r = 0.315) in our participants. We also reported that peak torque between diet groups was not significant (*p* = 0.074); furthermore, we reported that this statistic was further attenuated when gender was controlled. Had we controlled for gender, age, and energy intake, the resulting *p* value was similar to the unadjusted value (*p* = 0.078). We did not control for lean mass in this comparison between diet groups as our premise was that lean mass was a reflection of diet type. Indeed, peak torque per kilogram lean mass was virtually identical between diet groups. Tong et al. also reported a slight but significant reduction in strength for vegetarians versus omnivores from a large UK cohort (*n* = 229,806) and concluded that the differences in height, lean mass, and physical activity likely contributed to the difference in strength in vegetarians versus omnivores [14]. We were transparent in our analyses and followed all statistical assumptions, and we acknowledged in our report that additional research is necessary to comprehend the effect of protein quality on physical performance. 

We hope that these comments adequately address the concerns of Genoni et al. We value the opportunity to discuss our results in further detail. 
